# The influence of temperament and perinatal factors on language development: a longitudinal study

**DOI:** 10.3389/fpsyg.2024.1375353

**Published:** 2024-07-04

**Authors:** Andrea Balázs, Krisztina Lakatos, Veronika Harmati-Pap, Ildikó Tóth, Bence Kas

**Affiliations:** ^1^Institute for General and Hungarian Linguistics, HUN-REN Hungarian Research Centre for Linguistics, Budapest, Hungary; ^2^Sound and Speech Perception Research Group, Institute of Cognitive Neuroscience and Psychology, HUN-REN Research Centre for Natural Sciences, Budapest, Hungary; ^3^Department of Cognitive Science, Faculty of Natural Sciences, Budapest University of Technology and Economics, Budapest, Hungary; ^4^MTA-ELTE Language-Learning Disorders Research Group, Eötvös Loránd University, Bárczi Gusztáv Faculty of Special Needs Education, Budapest, Hungary

**Keywords:** language development, temperament, gestational age, longitudinal study, sex differences

## Abstract

Early language development is characterized by large individual variation. Several factors were proposed to contribute to individual pathways of language acquisition in infancy and childhood. One of the biologically based explaining factors is temperament, however, the exact contributions and the timing of the effects merits further research. Pre-term status, infant sex, and environmental factors such as maternal education and maternal language are also involved. Our study aimed to investigate the longitudinal relationship between infant temperament and early language development, also considering infant gender, gestational age, and birthweight. Early temperament was assessed at 6, 9, 18, 24, and 30 months with the Very Short Form of Infant Behavior Questionnaire (IBQ-R) and the Very Short Form of Early Childhood Behavior Questionnaire (ECBQ). Early nonverbal communication skills, receptive and expressive vocabulary were evaluated with the Hungarian version of The MacArthur Communicative Development Inventory (HCDI). Our study adds further evidence to the contribution of infant temperament to early language development. Temperament, infant gender, and gestational age were associated with language development in infancy. Infants and toddlers with higher Surgency might enter communicative situations more readily and show more engagement with adult social partners, which is favorable for communication development. Gestational age was previously identified as a predictor for language in preterm infants. Our results extend this association to the later and narrower gestational age time window of term deliveries. Infants born after longer gestation develop better expressive vocabulary in toddlerhood. Gestational age may mark prenatal developmental processes that may exert influence on the development of verbal communication at later ages.

## 1 Introduction

Early language development shows great individual variation both in the extension and the expansion of receptive and expressive vocabulary. The range of a 12-month-old, typically developing child’s receptive vocabulary may span from 25 to more than 200 words, and similar variation can be observed in expressive vocabulary (Fenson et al., 2007). Some children utter their first words by the age of 12 months while others only after 18 months. The rate of development begins to even out by the third year of life (Fenson et al., 2007). Previous studies indicated several biological factors influencing the dynamics of linguistic development in early childhood. Gestational age (Barre et al., 2011), birthweight (Stolt et al., 2009), infant sex (Law et al., 2019) and infant temperament (Ishikawa-Omori et al., 2022) were among these factors, however, social class, family history, certain environmental characteristics (AlHammadi, 2017) seem to play a role.

Concerning gestational age, studies suggest that children born very preterm and extremely preterm exhibit delayed language skills compared to full-term children (Foster-Cohen et al., 2007, 2010). In their earlier work, Foster-Cohen et al. (2007) studied 90 preterm children (*N* = 36 extremely preterm gestational age < 28 weeks, and *N* = 54 very preterm, gestational age 28–33) and 102 full-term children (gestational age 38–41). The MacArthur-Bates Communicative Development Inventory: Words and Sentences (CDI-WS) at 2 years of corrected age was associated with gestational age at birth. Vocabulary size, word use quality, morphological and syntactic complexity were related to longer gestation before birth. An association between gestational age and language outcomes persisted after the authors controlled for child and family factors otherwise related to gestational age. At 4 years (Foster-Cohen et al., 2010), the association between language development and very preterm birth was replicated. These children had significantly poorer linguistic outcomes even after excluding children with neurosensory impairment and statistical control for the effect of social risk. By contrast, Pérez-Pereira et al. (2016) studied language performance at 30 months with the Galician version of the CDI. Comparing low-risk preterm (mean gestational age, GA: 32.60 weeks) and full-term children (GA: 39.84 weeks), they found no significant differences in the language outcomes: word production, MLU and sentence complexity between groups.

However, the third trimester is characterized by important developmental changes in the brain. Shortened gestation, even within the normal term delivery range (greater than 37 weeks), had long-lasting effects on neural development in a healthy, low-risk population (Davis et al., 2011) with lower gray matter density detected by magnetic resonance imaging. These structural differences may lead to variation in later cognitive development as well. Can et al. (2013) identified several brain regions with early white matter and gray-matter concentrations in association with infants’ receptive language ability and expressive language at 12 months. The indicated cerebellum, PLIC/cerebral peduncle, and hippocampus are suggested to be associated with early language development. These brain developmental processes may contribute to the underlying mechanism connecting higher gestational age with better receptive language at 24 months of age in a sample of toddlers born after 32 weeks of gestation (Snijders et al., 2020). The Norwegian Mother and Child Cohort Study found that children born both early-term and late-preterm had an increased risk for communication impairment at 18 months and for expressive language impairments at 36 months (Stene-Larsen et al., 2014). Thus, we hypothesize that the linguistic performance of a full-term child may also be related to gestational age in a middle-class, term infant sample.

The contribution of birthweight to variations in language development tends to be confounded by preterm status (Stolt et al., 2009; Barre et al., 2011). No effect of birthweight on language outcomes was detected in a sample of Hungarian children on the Hungarian version of CDI-III (Dale et al., 2001; Fenson et al., 2007; Kas et al., 2022) at 2–4 years of age. The sample of 1,424 term children included 9.3% low-birthweight (<2,500 g) children. CDI scores were predicted by children’s age, gender, and parents’ education level, whereas other factors including birthweight, birth problems, number of siblings, birth order, multilingualism, familial net income, and children’s chronic illness did not have significant effects. Individual differences within normal birthweight (>2,500 g) have not yet been linked to language development, however, Full-scale IQ performance was positively associated with birthweight within the normal range (Matte et al., 2001). Marinopoulou et al. (2021) found that the number of words used by children at age 2.5 years was associated with deficits in intellectual functioning at age 7 years. Children who used 50 words or fewer at age 2.5 years had lower scores of Full-scale IQ, verbal comprehension, working memory, and perceptual reasoning at age 7 years. Given the contradictory results and the potential association via IQ, further investigation of the role of birthweight is needed.

Although there is a growing body of research on the role of infant temperament (Ishikawa-Omori et al., 2022), the results are inconclusive. Studies differ in the definitions of temperament, the stage of language development investigated, the age range of the children, the length of data collection, and the set of other variables included in the analyses. The diversity of these parameters makes it difficult to compare the results. Major theories agree that temperament is inherently present at an early age and influences the expression of behaviors related to activity, affectivity, and self-regulation (Goldsmith et al., 1987; Shiner et al., 2012). However, different approaches to temperament use divergent operational definitions and thus operate within somewhat different frameworks. According to Rothbart and Derryberry (1981) and Rothbart (2007), whose approach was applied in the present study, temperament is constitutionally based, can be measured from infancy, and shows a relatively stable pattern extending over the lifetime (Hampson and Goldberg, 2006; Putnam et al., 2008; Kopala-Sibley et al., 2018; Tang et al., 2020). It can be defined as individual differences in reactivity and self-regulation that manifest in emotions, activity, and attention. Temperament is described by 3 major, distinct factors: Positive Emotionality/Surgency, Negative Affectivity and Regulatory Capacity/Effortful Control (see Table 1 for example items assessing the three factors). Buss and Plomin’s (1984) approach shares some of the concepts and behaviors observed, Thomas and Chess (1977) defined rather different temperament types based on nine dimensions of temperament that captured patterns relevant to clinical practice. While these theories consider emotions and affectivity as components of temperament, Goldsmith (1996) sees temperament as the expression and regulation of emotions. Thus, instruments based on one theory or the other may capture different aspects of temperament.

**Table 1:** Example items assessing the 3 factors of temperament (effortful control, Surgency, negative affectivity).

	Very Short Form of Infant Behavior Questionnaire (IBQ-R)	Very Short Form of Early Childhood Behavior Questionnaire (ECBQ)
Surgency	During a peekaboo game, how often did the baby laugh?	When offered a choice of activities, how often did your child decide what to do very quickly and go after it?
	When hair was washed, how often did the baby vocalize?	When encountering a new activity, how often did your child get involved immediately?
	How often during the week did your baby move quickly toward new objects?	While participating in daily activities, how often did your child seem full of energy, even in the evening?
Effortful control	How often during the last week did the baby enjoy being read to?	When told “no,” how often did your child stop the forbidden activity?
	How often during the last week did the baby play with one toy or object for 5–10 min?	When asked to wait for a desirable item (such as ice cream), how often did your child wait patiently?
	How often during the last week did the baby stare at a mobile, crib bumper or picture for 5 min or longer?	When asked to do so, how often was your child able to be careful with something breakable?
Negative affectivity	When tired, how often did your baby show distress?	When visiting a new place, how often did your child not want to enter?
	When introduced to an unfamiliar adult, how often did the baby cling to a parent?	When told “no,” how often did your child become sadly tearful?
	When introduced to an unfamiliar adult, how often did the baby refuse to go to the unfamiliar person?	Following an exciting activity or event, how often did your child seem to feel down or blue?

Based on Rothbart’s concept, longitudinal positive associations were found between temperament and expressive language skills. Children’s expressive vocabulary and length of utterance at 24 months were associated with Approach and Perceptual Sensitivity measured at 8 and 12 months of age (Davison et al., 2019). The scales of Approach and Perceptual Sensitivity, along with others, contribute to the Surgency factor (Gartstein and Rothbart, 2003). Laake and Bridgett (2014) also reported that a higher Surgency score measured at 10 months was predictive of improved expressive but not receptive language at 14 months. This relationship might be related to higher infant Surgency predicting higher levels of toddler Effortful Control (Putnam et al., 2008), and in turn, Effortful Control was reported to be associated with expressive language (Bruce et al., 2022). Also, as Positive Anticipation contributes to the Surgency factor, the general learning enhancing aspect or/and the social aspect of positive affect might be considered here as well. Kort et al. (2001) reported that positive affect enhanced students’ learning behavior. Yang et al. (2013) found that positive affect was related to better working memory and had a weaker relationship with short-term memory. They suggest that positive affect facilitates controlled cognitive processing, leading to improved learning ability. We may assume that improved learning ability may support language learning as well. Language learning is greatly facilitated by interactions with social partners. Dixon and Smith (2000) claim that individual differences in positive or negative emotionality might moderate the willingness of social partners to enter social dialogs in the first place, thus influencing exposure to language. Ishikawa-Omori et al. (2022) studied receptive and expressive vocabulary at 40 months. They found that two scales contributing to the Negative Affectivity factor, Motor Activation and Perceptual Sensitivity at 18 months predicted language skills at 40 months, however, the associations pointed in opposite directions. Higher scores on Perceptual Sensitivity were related to larger expressive and receptive vocabulary at 40 months, while higher scores on Motor Activation were related to poorer receptive and expressive vocabulary. Garello et al. (2012) also found concurrent negative correlations between Motor Activation and language development in 24- and 30-month-old children.

Early attentional control and the capacity for self-regulation, which consistently loaded on the Effortful Control factor, were associated positively with language development in infancy and early childhood as well. Dixon and Shore (1997) and Dixon and Smith (2000) reported that attentional control, positive affect and emotional stability measured at 13 months predicted the efficiency of language acquisition, including the time of appearance of first words and the time and speed of vocabulary expansion at 20–21 months. Dixon and Smith (2000) explained this pattern of longitudinal association by Rothbart and Bates’s (2007) theory of an early attentional control system, which corresponds to the maturation of the anterior attentional system at the end of the first year. This early attentional control system allows children to voluntarily direct and maintain attention and allows flexibility in awareness. In fact, emergent control of attention indicated by increases in the duration of orientation from 7 to 10 months was found to be associated with advanced language production at 20 months.

In summary, higher Positive Affect and Effortful Control at the end of the first year and the beginning of the second year are associated with better language performance between 1 and 2 years of age. Conversely, a higher score on the Negative Affectivity between 18 and 30 months is associated with poorer language performance between 24 and 40 months. Additionally, Negative Affectivity may influence the rate of expressive language development around the age of 2 and beyond due to a lower likelihood of engaging in social interactions.

The present study focused on examining the role of perinatal variables in addition to temperament in language development and assessing concurrent and longitudinal relationships in a longitudinal design. Both language development and temperament were evaluated repeatedly, allowing for capturing the potentially changing patterns of associations between temperament and language skills. In addition to expressive language and receptive vocabulary, gestural communication was measured. According to the design of CDI, the latter two were assessed up to 18 months (Frank et al., 2021). Regression models were used to determine the effect of temperament, infant gender and perinatal factors.

## 2 Materials and methods

### 2.1 Participants

A longitudinal project on early language development was carried out by recruiting 186 families. The inclusion criteria for the present study were that the child was born on time (gestational age > 37 and birthweight >2,500) and was taken to the baby lab at least once. All infants included in the present investigation were of low social risk and were the first-born children of their mothers. As is common in developmental studies with voluntary participants, mothers with higher education were overrepresented, with 75% having college or university degrees. All participants came from metropolitan (Budapest) or agglomeration areas, all children were monolingual. No hearing problems were reported. The sample was ethnically homogeneous Caucasians of Hungarian origin. Families were recruited at the infant’s birth, 4, 9, and 18 months of age (see Table 2). All families received detailed information on the study, and informed consent was obtained. The first wave included 74 middle-class mothers recruited in the HONVED PMC hospital’s maternity ward. Data were collected up to 18 months in this phase. An additional recruitment at 18 months was planned to increase the sample size continuing to the second phase, however, as the dropout rate was higher than previously expected due to the COVID-19 epidemic, additional recruitment of 4- and 9-month-old infants was carried out (see Table 2). The sex ratio and infant characteristics in the participating and the dropout families did not differ significantly. The present data set includes varying numbers of infants at different ages due to the disruption caused by the pandemic breaking out during data collection and preventing families from visiting the child laboratory. The exact numbers of available data at each age are presented in Table 2.

**Table 2:** Number of participants at each data collection point in cohorts recruited at different ages.

Data collection points	Cohorts				Total number of participants
	Newborn	4-month	9-month	18-month	
6 months	68	39	-	-	107
9 months	56	39	26	-	121
18 months	53	36	25	37	151
24 months	42	35	25	37	139
30 months	40	35	25	31	131

### 2.2 Procedures and instruments

According to the original protocol, mothers were to fill in the questionnaires in the baby lab while lab assistants played with the children and administered tests in the passive presence of the mother. However, the Covid-19 pandemic resulted in some mothers completing the questionnaires from their homes online. There were no significant differences in temperament or language between the questionnaires administered before and during the pandemic.

Ethical approval for the study was granted by ETT-TUKEB (1942-12/2016) and EPKEB (77/2015).

#### 2.2.1 Measurements of child temperament

Infant temperament was assessed using the Very Short Forms of the Infant Behavior Questionnaire (IBQ–R) (Putnam et al., 2014) and the Early Childhood Behavior Questionnaire (ECBQ) (Putnam et al., 2006). Mothers completed the 37-item IBQ–R and 36-item ECBQ (Hungarian versions: Lakatos et al., 2010) either in the baby lab or online at home. The IBQ–R was administered at 6 and 9 months of infant’s age, whereas the ECBQ was at 18, 24 and 30 months. Mothers rated the frequency of their infants’ behaviors over the past two weeks using seven-point Likert scales. Three main factors were computed: Surgency, Effortful Control, and Negative Affectivity. Cronbach alpha coefficients of internal consistency for these factors in this sample were between 0.607 and 0.805 (see Table 3). Missing data were not substituted.

**Table 3:** Cronbach’s alpha coefficients of internal consistency of IBQ-R-SF and ECBQ-SF factors.

Age Scale name	Cronbach’s Alpha
6 months	
Surgency	0.686
Effortful control	0.677
Negative affectivity	0.805
9 months	
Surgency	0.607
Effortful control	0.705
Negative affectivity	0.756
18 months	
Surgency	0.738
Effortful control	0.703
Negative affectivity	0.688
24 months	
Surgency	0.680
Effortful control	0.723
Negative affectivity	0.626

#### 2.2.2 Measurements of language and communication skills

**Figure 1:**
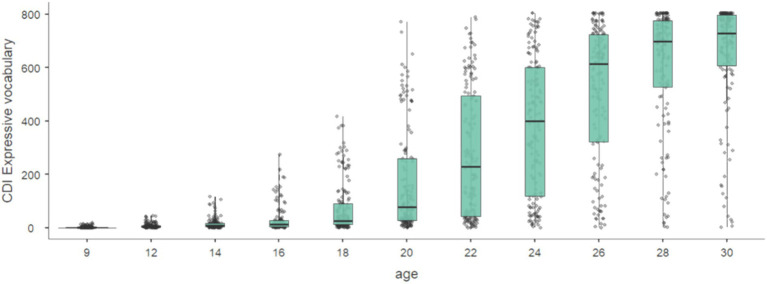
CDI expressive vocabulary scores by age.

For the assessment of early language development, the Hungarian adaptation of the MacArthur-Bates Communicative Development Inventory (CDI) Words & Gestures and Words & Sentences parent report forms (Fenson et al., 2007) has been used (Kas et al., 2010, 2022). This questionnaire relies on maternal (caregiver) reports to explore children’s receptive and expressive vocabulary and assess their level of speech comprehension, gesture use, morpheme acquisition, and syntactic complexity through systematic questions. CDI forms are suitable for assessing language development in typically developing children aged 8–30 months or older with developmental disorders. The present study considers the following CDI variables: (1) receptive vocabulary total score, (2) expressive vocabulary total score, and (3) gestures total score including sub-scores of object manipulation, imitation of adults, symbolic activity, and non-verbal gesture use. The CDI was first administered at 9 months of age, followed by a second administration at 12 months. Thereafter, the course of language development was monitored at two-month intervals until the age of 30 months (Figure 1). For the present report, language data from 18, 24, and 30 months was included in the analyses. Eighteen months of age represents a major turning point in language development, as this is the last age when all 3 dimensions of the CDI (receptivity, expression and gesture) are assessed. Twenty-four-month language data was included because it showed the highest variability. Thirty-month expressive language as measured by CDI was also characterized by good variability. Temperament was also assessed at these ages, thus concurrent associations can be examined.

#### 2.2.3 Analyses

Data were analyzed with IBM SPSS (Version 26). Descriptive measures of linguistic variables obtained from H-CDI (Receptive and Expressive Vocabulary and Communicative Gestures) and of the temperament variables obtained from IBQ-R and ECBQ (Surgency, Effortful Control, Negative Affectivity), perinatal variables, and infant sex were calculated (Table 4). According to the results of Shapiro–Wilk tests, parametric and non-parametric tests were carried out in the analyses. Sex differences were investigated for all variables. To examine the contribution of temperament, perinatal factors to the individual variation in language development, we first analyzed correlations of these variables with language development at various ages (see Table 5). Variables with significant associations were entered as predictors in stepwise linear regression analysis to determine their predictive value on the dependent linguistic variables, such as receptive and expressive vocabulary and communicative gestures at 18 months of age, and expressive vocabulary at 24 and 30 months of age.

**Table 4:** Descriptive statistics of and differences by infant sex in perinatal factors, temperament scales, and language skills.

	Variables at different ages	*N*	Mean (SD)	Range Min-Max	Girls *N*	Girls Mean (SD)	Boys Mean (SD)	*H or F* statistic
	Birthweight	171	3416.46 (405.50)	2,410–5,350	82	3278.51 (358.18)	3,543 (406.80)	**20.327** ^*^
	Gestational age	171	39.56 (1.128)	37–43	81	39.54 (1.15)	39.56 (1.11)	0.242
Surgency	6 months	105	5.26 (0.69)	3.75–6.72	53	5.18 (0.76)	5.34 (0.59)	1.427
	9 months	121	5.31 (0.65)	3.77–6.75	61	5.21 (0.65)	5.41 (0.64)	**2.891** ^**+**^
	18 months	140	5.26 (0.76)	2.83–6.58	69	5.15 (0.79)	5.37 (0.72)	2.644
	24 months	130	5.32 (0.73)	3.00–6.67	63	5.28 (0.68)	5.35 (0.77)	0.466
Effortful control	6 months	105	5.43 (3.54)	3.5–6.98	53	5.47 (0.648)	5.39 (0.66)	0.452
	9 months	121	5.28 (3.98)	2.75–6.73	61	5.30 (0.63)	5.23 (0.77)	0.010
	18 months	140	4.65 (2.77)	2.455–6.42	69	4.73 (0.75)	4.57 (0.71)	1.673
	24 months	130	4.91 (3.00)	2.75–6.83	63	4.95 (0.67)	4.88 (0.75)	0.322
Negative affectivity	6 months	105	3.54 (0.96)	1.25–6.20	53	3.64 (1.03)	3.44 (0.88)	1.104
	9 months	121	3.98 (0.90)	1.25–6.00	61	4.03 (0.97)	3.94 (0.83)	0.289
	18 months	140	2.77 (0.71)	1.29–4.91	69	2.70 (0.69)	2.84 (0.72)	1.349
	24 months	130	3 (0.73)	1.50–5.91	63	3.02 (0.80)	2.98 (0.66)	0.011
Language and communication	Receptive vocabulary 18 months	147	311.45 (100.25)	27–455	73	326.15 (95.17)	296.95 (103.62)	**2.939** ^**+**^
	Gestures 18 months	147	57.99 (11.41)	25–81	73	61 (11.26)	55.03 (10.83)	**10.751** ^**^
	Expressive vocabulary 18 months	147	72.66 (95.35)	0–383	73	84.60 (105.34)	60.88 (83.40)	**5.043** ^*^
	Expressive vocabulary 24 months	144	380.60 (256.98)	1–804	71	430.23 (237.59)	332.34 (267.38)	**5.097** ^*^
	Expressive vocabulary 30 months	138	655.32 (205.14)	2–804	69	691.16 (183.36)	619.58 (220.39)	**5.652** ^*^

+*p* < 0.10, *p < 0.05, **p < 0.01, ***p < 0.001. Bold highlights significant sex differences.

**Table 5:** Correlations between perinatal factors, temperament, and language.

	Receptive vocabulary	Gestures	Expressive vocabulary		
	18 months	18 months	18 months	24 months	30 months
Birthweight	0.204^*^ **(146)**	0.044 (146)	**0.153**^**+**^ **(146)**	0.130 (144)	0.179^*^ **(138)**
Gestational age	0.153 (145)	**0.161**^**+**^ **(145)**	0.265^**^ **(145)**	0.212^*^ **(143)**	**0.167**^**+**^ **(137)**
Temperament					
Surgency					
6 months	0.110 (82)	**0.200**^**+**^ **(82)**	−0.034 (82)	−0.009 (78)	−0.022 (75)
9 months	. 285^**^ **(106)**	0.233^*^ **(106)**	**0.165**^**+**^ **(106)**	0.149 (102)	**0.183**^**+**^ **(98)**
18 months	0.285^***^ **(137)**	0.319^***^ **(137)**	0.191^*^ **(137)**	0.183^*^ **(133)**	−0.137 (128)
24 months				0.213^*^ **(130)**	0.200^*^ **(126)**
30 month				**0.064**^**+**^ **(114)**	
Effortful control					
6 months	0.089 (82)	**0.190**^**+**^ **(82)**	0.089 (82)	0.022 (78)	0.016 (75)
9 months	0.221^*^ **(106)**	0.221^*^ **(106)**	0.122 (106)	0.026 (102)	0.088 (98)
18 months	**0.160**^**+**^ **(137)**	0.313^***^ **(137)**	0.137 (137)	0.122 (133)	**0.152**^**+**^ **(128)**
24 months				0.096 (130)	0.133 (126)
30 month				0.183 (114)	
Negative affectivity					
6 months	0.169 (82)	0.0147 (82)	0.077 (82)	0.187 (78)	0.233^*^ **(75)**
9 months	0.062 (106)	0.032 (106)	0.070 (106)	0.105 (102)	0.095 (98)
18 months	0.031 (137)	−0.138 (137)	−0.061 (137)	−0.202^*^ **(133)**	**−0.159**^**+**^ **(128)**
24 months				−0.021 (130)	−0.024 (126)
30 month					−0.005 (114)

Pearson’s r or Spearman’s rho, +*p* < 0.10, *p < 0.05, **p < 0.01, ***p < 0.001. Bold: highlights significant and trend-level correlations.

## 3 Results

### 3.1 Descriptive statistics

Means and standard deviations for the whole sample, and for boys and girls separately are presented in Table 4. The age-related growth of expressive vocabulary between 9 and 30 months is depicted in Figure 1.

One-way ANOVA or Kruskal-Wallis tests were applied to assess sex differences. No gender differences were observed in gestational age, but boys were significantly heavier at birth [*H*(1, *n =* 179] = 20.394, *p* < 0.001). Likewise, no significant differences between boys and girls appeared on temperament scales at any age, apart from a statistical trend towards boys scoring higher on Surgency at 9 months [*F*(1,119) = 2.891, *p* = 0.092]. However, significant sex differences were found in CDI language scores (Table 4). Girls scored higher on all CDI sub-scales at most time points, except for receptive vocabulary at 18 months [*H*(1, *n* = 147] = 2.939, *p* = 0.086), yet with a tendency in favor of girls.

### 3.2 Correlations with language scores

Bivariate relationships between language development and temperament, demographic and perinatal variables were explored by correlation analyses (Table 5) to select variables for regression analyses predicting language outcomes.

Concurrent correlations were investigated at 18, 24 and 30 months. 18-month Surgency showed a consistent relationship with all measures of language development: higher Surgency was related to better language skills. Better Effortful Control was significantly related to more developed use of gestures and there was a tendency toward better receptive vocabulary. At 24 months, higher Surgency was related to better expressive vocabulary. At 30 months, the association between these two measures only showed a trend-level correlation, however, in the same direction as at earlier ages.

Longitudinal correlations were weak and sparse, however, Surgency at various ages tended to be related to measures of language and communicative development at later ages. Higher Surgency at 9 months was related to higher receptive vocabulary and gesture use at 18 months. Similarly, positive associations appeared between 18-month Surgency and expressive vocabulary at 24 months, and 24-month Surgency and expressive vocabulary at 30 months, with higher Surgency being related to a more extensive expressive vocabulary. In addition, 18-month receptive vocabulary and gesture use were associated with 9-month Effortful Control. Higher Negative Affectivity at 6 months was significantly correlated with more developed expressive vocabulary at 24 months. However, later Negative Affectivity (at 18 months) had the opposite relationship with expressive vocabulary at 30 months: more negative affect was associated with lower expressive vocabulary a year later.

Of the temperament factors in infancy and early childhood, Surgency seems to be indicated in language acquisition both concurrently and longitudinally, spanning from receptive to expressive language.

### 3.3 Longitudinal predictors of language development

To better understand how temperament and perinatal factors affect each language and communication skill measured by the H-CDI, we conducted linear regression analyses with stepwise selection separately for each CDI variable at 18, 24 and 30 months. Temperament variables of preceding ages, perinatal variables, and infant sex showing significant correlations with the predicted variable were included in the regression.

First, we examined receptive vocabulary at 18 months (Table 6). Birthweight, Surgency and Effortful Control at 9 months were entered in the model. In the final model [*R*^2=^0.089, *F*(1,104) = 10.161, *p* = 0.002], the single significant predictor was Surgency measured at 9 months (*β* = 0.298, *p* = 0.002).^1^

**Table 6:** Receptive vocabulary at 18 months was predicted by 9-month Surgency.

Predictors	Beta	Sig.	_R_2	Change in R^2^	Change in F	Sig. change in F	F	df2	Sig.
Model 1			0.089	0.089	10.161	0.002	10.161	104	0.002
Surgency (9 months)	0.298	0.002							
				0.044	5.223	0.024	7.899	103	0.001

Stepwise linear regression analyses, *N* = 106, excluded variables birthweight, 9-month Effortful Control.

In the model predicting gestures at 18 months, sex, Surgency, and Effortful Control at 9 months were entered (see Table 7). In the final model [R^2=^0.111, *F*(2,103) = 7.559, *p* = 0.001], predictive variables were Surgency measured at 9 months (*β* = 0.272, *p* = 0.004) and sex (*β* = −0.275, *p* = 0.004).^2^

**Table 7:** Gestures at 18 months were predicted by infant sex and Surgency (9 months).

Predictors	Beta	Sig.	_R_^2^	Change in R^2^	Change in F	Sig. change in F	F	df2	Sig.
Model 1			0.056	0.056	6.125	0.001	6.125	104	0.015
Sex	−0.236	0.015							
Model 2			0.128	0.072	8.548	0.013	7.559	103	0.001
Sex	−0.275	0.004							
Surgency (9 months)	0.272	0.004							

Stepwise linear regression analyses, *N* = 106, excluded variables: effortful control (9 months).

To predict expressive vocabulary at 18 months, only sex and gestational age were entered, as no temperament variable showed a significant correlation with this language outcome (see Table 8). Here, only one model was generated [R^2=^0.076, *F*(1,143) = 11.778, *p* < 0.001], with gestational age reaching significance (*β* = 0.276, *p* < 0.001).^3^

**Table 8:** Expressive vocabulary at 18 months was predicted by gestational age.

Predicators	Beta	Sig.	_R_2	Change in R^2^	Change in F	Sig. change in F	F	df2	Sig.
Model 1			0.076	0.076	11.778	0.001	11.778	143	0.001
Gestational age	0.276	0.001							

Stepwise linear regression analyses, *N* = 145, excluded variables: sex.

Table 9 presents the regression model predicting expressive vocabulary at 24 months, in which sex, gestational age, Surgency and Negative Affectivity at 18 months were entered. In the final model [R^2=^0.112, *F*(2,129) = 8.163, *p* < 0.001], gestational age (*β* = 0.248, *p* = 0.004) and Negative Affectivity measured at 18 months (*β* = −0.199, *p* = 0.019) were the significant predictors.^4^

**Table 9:** Expressive vocabulary at 24 months was predicted by gestational age and negative affectivity (18 months).

Predictors	Beta	Sig.	_R_2	Change in R^2^	Change in F	Sig. change in F	F	df2	Sig
Model 1			0.073	0.73	10.298	0.002	10.298	130	0.002
Gestational age	0.271	0.002							
Model 2			112	0.39	5.660	0.019	8.163	129	0.000
Gestational age	248	0.004							
Negative Affectivity (18 months)	−0.199	0.019							

Stepwise linear regression analyses, *N* = 132, excluded variables: sex, Surgency (18 months).

Infant sex, birthweight, Negative Affectivity at 6 months, and Surgency at 24 months were entered into the regression to predict expressive vocabulary at 30 months (see Table 10). In the final model [R^2=^0.088, *F*(1,64) = 6.152, *p* = 0.016], the only significant contributor was Surgency at 24 months^5^ (*β* = 0.296, *p* = 0.016).

**Table 10:** Expressive vocabulary at 30 months was predicted by Surgency at 24 month.

Predictors	Beta	Sig.	_R_2	Change in R^2^	Change in F	Sig. change in F	F	df2	Sig.
Model 1			0.088	0.088	6.152	0.003	6.15	64	0.016
Surgency (24 months)	0.296	0.016							

Stepwise linear regression analyses, *N* = 66, excluded variables: sex, birthweight, negative affectivity (6 months).

## 4 Discussion

Our study aimed to investigate the longitudinal relationship between infant temperament and early language development, also considering infant sex and gestational age. Several data collection points for temperament (6–30 months) and language development (18–30 months) were included. Our findings support the role of both infant temperament and perinatal factors in early language development. Nine-month Surgency forecasted receptive vocabulary at 18 months and also contributed to gestural communication at 18 months in addition to infant sex. Gestational age predicted expressive vocabulary at 18 and 24 months. In addition, Negative Affectivity at 18 months also contributed to 24-month expressive vocabulary. Thirty-month expressive vocabulary was predicted by Surgency measured at 24 months.

While Surgency appears to have a significant influence on receptive language and gestures at 18 months, and expressive vocabulary at 30 months, there was a lack of association with expressive vocabulary at 18 and 24 months. Instead, expressive vocabulary at these ages was related to gestational age. Thus, there seems to be a discontinuity in the effect of Surgency, with the emergence of gestational age. Bates et al. (1992) describe increases in vocabulary and grammar along with increases in synaptic density and brain metabolism between the ages of 16–30 months. These brain developmental processes might not be independent of prenatal brain development potentially marked by gestational age. This may be reflected in gestational age predicting 18- and 24-month expressive vocabulary. Surgency, however, may play a role in the expansion of gestural and verbal communication via potentially increased exposure to communicative signals and engagement in social interaction (Laake and Bridgett, 2014). This may be reflected in the association with a more extensive receptive vocabulary and gestures at 18 months, and expressive language at a later age (30 months), when verbal communication is established in most of the children.

### 4.1 Surgency

Several studies have linked positive affectivity with language development in infancy and early childhood (Laake and Bridgett, 2014; Pérez-Pereira et al., 2016; Davison et al., 2019). Positive affectivity contributes to the Surgency factor in Rothbart’s temperament model (Gartstein and Rothbart, 2003). Laake and Bridgett found that 10-month-old infants with higher Positive Affectivity/Surgency, as measured by IBQ-R, showed improved expressive language at 14 months. Davidson’s study also supported these findings, as infant Positive Affectivity/Surgency measured at 8 and 12 months predicted expressive language skills at 24 months.

Consistent with the literature, we also found Surgency to be related to early language skills. Surgency at 9 months predicted receptive vocabulary and gesture use at 18 months, while Surgency measured at 24 months was a significant contributor to expressive vocabulary at 30 months. Of concurrent associations between Surgency and language measures, only correlations with 18-month receptive vocabulary and gesture use remained significant after Bonferroni correction. However, at least a trend-level association with concurrent Surgency pointing in the same direction can be observed for expressive vocabulary at all ages. Thus, infants with higher Surgency scores demonstrated better language abilities, both in terms of receptive and expressive language. These results suggest that Surgency may be related to language development over an extended period. Since there is some stability in Surgency over time (correlations among Surgency values measured between 9–30 months ranged between 0.358–0.694), temperament can be expected to show a weak longitudinal correlation with expressive communication.

As children with high Positive Affectivity/Surgency are more likely to engage in and elicit social interactions, they have more opportunities to practice and improve their expressive language skills (Laake and Bridgett, 2014). This assumption could also apply to gesture use and receptive language, as both are related to expressive language use. Extensive social interactions provide more opportunities not only for the use of expressive vocabulary but also for gestural communication. More social interactions may result in varied, and increased amounts of language stimuli, fostering the development of language skills.

### 4.2 Effortful control

In our study, Effortful Control was not a significant predictor of language development in the regression models. Only weak correlations were observed between Effortful Control at 9 months and gesture use and receptive vocabulary at 18 months. Medium concurrent correlation with gesture use was also observed at 18 months.

The link between effortful control and language development remains unclear, despite some studies (Salley and Dixon, 2007; Keller et al., 2016) suggesting a positive relationship that could potentially be attributed to varying attentional capacities, which are thought to support language acquisition (Snijders et al., 2020). Effortful Control, as measured by Rothbart’s temperament questionnaires, is related to the functioning of the executive network (Posner et al., 2016). In turn, a link was demonstrated between the executive network and language development, production, and comprehension (Ye and Zhou, 2009; Shokrkon and Nicoladis, 2022). Furthermore, language development may also contribute to executive function development and self-regulation (Roben et al., 2013; Bruce et al., 2023).

However, Bruce et al. (2022) found that Effortful Control was only related to concurrent language, and 10-month Orienting/Effortful Control did not predict 24-month expressive language. Similarly, Ishikawa-Omori et al. (2022) did not find a predictive link between Effortful Control at 18 months and language development at 40 months. Keller et al. (2016) only demonstrated a significant relationship in the second language competence of dual language learners in childhood. The lack of predictive power of Effortful Control preceding the age of language assessment in the regression models and the separately observed concurrent correlation are in line with these results.

### 4.3 Negative affectivity

Negative Affectivity was entered in regressions at 24 and 30 months, however, only 18-month Negative Affectivity proved to be a significant predictor for lower expressive vocabulary at 24 months. This result supports earlier findings that Negative Affectivity may be associated with worse language skills (Dixon and Smith, 2000; Garello et al., 2012; Ishikawa-Omori et al., 2022). For instance, Garello et al. found that at the ages of 24–30 months, increased Negative Emotionality and Motor Activity correlated with poorer language production and comprehension. Similarly, Ishikawa-Omori et al. (2022) reported that Motor Activity, a scale of the Negative Affectivity factor, measured at 18 months, predicted lower expressive and receptive language skills at 40 months. They suggested that fidgeting behavior may reduce the availability of attentional resources, and as a result, it could hinder language learning. Excessive negative emotions could limit the resources children can allocate for information processing and language learning. They may also influence the way the children and their social partners interact. Children displaying more negative affect indeed performed worse on a joint attention task at 21 months (Salley and Dixon, 2007).

### 4.4 Gestational age

Gestational age proved to be a significant predictor for expressive vocabulary at 18 and 24 months. It’s been well-documented that both preterm birth and low birthweight can negatively impact language development into school age and beyond (Husby et al., 2023). Emerging findings, however, suggest variation in the development of term-born children, indicating differing developmental trajectories for early-term, full-term, late-term and post-term children (MacKay et al., 2010; Espel et al., 2014; Bentley et al., 2016; Snijders et al., 2020; Dhamrait et al., 2021). Our results suggest that longer *in-utero* development may support the development of expressive language. The final weeks of intrauterine development are characterized by rapid brain development. Children born early-term will not benefit from the effect of uterine neurosteroids (Hüppi et al., 1998; Limperopoulos et al., 2005; Shaw et al., 2019) as long as children born at later gestational ages. Increasing evidence shows long-lasting brain structure differences in preterm infants (Inder et al., 2005; Rogers et al., 2018). For instance, variations in functional connectivity were present even in adolescence after preterm birth, suggesting distinctive neurodevelopment potentially underlying behavioral differences (Lubsen et al., 2011). Term-born infants’ brain development also seems to benefit from longer gestation, within the time window of 37–41st weeks. Gestational age was related to differences in brain development in school-age children (Davis et al., 2011; Nivins et al., 2023). Such a variation may contribute to the observed differences in cognitive functioning and language skills (Ma et al., 2022).

### 4.5 Infant sex

Other than temperament and gestational age, infant sex also seems to contribute to variations in language skills. Previous studies have shown sex differences in language acquisition (Eriksson et al., 2012; Law et al., 2019), which aligns with our findings. Except for receptive vocabulary at 18 months, girls performed significantly better on all language measures and infant sex predicted the use of gestures at 18 months. Although we have only assessed gestures at 18 months, previous studies found girls using more gestures and starting earlier than boys (Özçalişkan and Goldin-Meadow, 2010; Germain et al., 2022).

### 4.6 Conclusion

Our aim was to investigate the role of temperament and some perinatal and maternal characteristics on early language development in a sample of low-social-risk, first-born term infants. Our sample was rather homogeneous as all participants were Caucasian of Hungarian origin, and the maternal education level was generally high across the sample. Results indicate the contribution of Surgency both concurrently and longitudinally on various measures of language development and the influence of gestational age on expressive vocabulary at 18 and 24 months. Negative Affectivity only predicted expressive vocabulary at 24 months. Despite Effortful Control being correlated with 18-month language, it was not a significant predictor in the regression models.

However, a major limitation of the study was the sample size and the missing data due to the pandemic. The COVID-19 pandemic hit during data collection and caused unexpected loss of data (data collection could not be conducted due to closures) and thus a higher dropout rate. Although the pandemic had no direct influence on the data presented (no differences were observed on any measures between data collected pre-pandemic and pandemic, post-pandemic periods), there might be hidden underlying effects of the quarantine period. Only 3 families reported contracting COVID-19 during the data collection. Thus, we may assume that results were not influenced by the neurological effects of the viral infection. Another limitation was the relatively low reliability of some temperament factors (Surgency at 9 months: 0.61, Negative Affectivity at 24 months: 0.63). Since correlations of the Surgency factor were consistent with those of other ages (albeit weak across the board), we have decided to include it in the regression analyses.

Our results extend previous findings as we have demonstrated associations with Surgency at the early stages of language acquisition for both receptive and expressive vocabulary, and showed the additional significant contribution of gestational age and Negative Affectivity. Gestational age was identified as a predictor for language in preterm infants previously. Our results extend this association to the narrower time window of gestational age of full-term infants. The latter finding may have relevance for medical practice and child educational support agencies. In line with other studies highlighting difficulties in later academic performance, this calls for increased attention to the early development of early-term and term infants.

### 4.7 Future directions

Our results highlight the importance of longitudinal studies using tools to measure temperament based on the same theoretical concept over time. Also, investigating the small differences in gestational age in term infants in a larger sample may reveal important effects on language acquisition. With more evidence on how early-term status may influence later cognitive and language development, research on how certain environmental factors, such as socioeconomic status, maternal education, and quality of mother–child interaction might interact with gestational age can yield important results that can be translated into practices supporting early childhood development.

Extending the study beyond 30 months is crucial to identify early characteristics of developmental pathways leading to language impairment. The role of Surgency and the relative lack of power for Effortful Control in this sample calls for experimental investigation of the development of very early executive functions and attentional functioning.

## Data availability statement

The raw data supporting the conclusions of this article will be made available by the authors, without undue reservation.

## Ethics statement

The studies involving humans were approved by Medical Research Council, Scientific and Research Ethics Committee (ETT-TUKEB) United Ethical Review Committee for Research in Psychology (EPKEB). The studies were conducted in accordance with the local legislation and institutional requirements. Written informed consent for participation in this study was provided by the participants’ legal guardians/next of kin.

## Author contributions

AB: Formal analysis, Investigation, Project administration, Writing – original draft, Writing – review & editing, Data curation. KL: Conceptualization, Data curation, Formal analysis, Investigation, Methodology, Supervision, Writing – original draft, Writing – review & editing. VH-P: Investigation, Writing – review & editing. IT: Data curation, Investigation, Project administration, Writing – review & editing. BK: Conceptualization, Data curation, Formal analysis, Funding acquisition, Methodology, Supervision, Writing – review & editing.

## Footnotes

^1^Multicollinearity was not detected (birthweight, Tolerance = 0.99, VIF = 1.00; Surgency at 9 months, Tolerance = 1.00 VIF = 1.00; Effortful Control at 9 months, Tolerance = 0.84, VIF = 1.19).

^2^Multicollinearity was not detected (sex, Tolerance = 0.98, VIF = 1.02; Surgency at 9 months, Tolerance = 0.98, VIF = 1.02; Effortful Control at 9 months, Tolerance = 0.83, VIF = 1.21).

^3^Multicollinearity was not detected (gestational age, Tolerance = 1.00, VIF = 1.00; sex, Tolerance = 1.00, VIF = 1.00).

^4^Multicollinearity was not detected (gestational age, Tolerance = 0.98, VIF = 1.01; Negative Affectivity at 18 months, Tolerance = 0.98, VIF = 1.01; sex, Tolerance = 0.98, VIF = 1.01; Surgency at 18 months, Tolerance = 0.97, VIF = 1.03).

^5^Multicollinearity was not detected (Surgency at 24 months, Tolerance = 1.00, VIF = 1.00; sex, Tolerance = 0.98, VIF = 1.02; birthweight, Tolerance = 0.99, VIF = 1.00; Negative Affectivity at 6 months, Tolerance = 0.98 VIF = 1.02).

## References

[ref1] AlHammadi F. S. (2017). Prediction of child language development: A review of literature in early childhood communication disorders. Lingua 199, 27–35. doi: 10.1016/j.lingua.2017.07.007

[ref2] Barre N. Morgan A. Doyle L. W. Anderson P. J. (2011). Language abilities in children who were very preterm and/or very low birthweight: a meta-analysis. J. Pediatr. 158, 766–774.e1. doi: 10.1016/j.jpeds.2010.10.032 21146182

[ref3] Bates E. Thal D. Janowsky J. S. (1992). Early language development and its neural correlates. In Handbook of neuropsychology. (eds.) Segalowitz S. J. Rapin I. Vol. 7. Child neuropsychology. Amsterdam: Elsevier Science Publishers.

[ref4] Bentley J. P. Roberts C. L. Bowen J. R. Martin A. J. Morris J. M. Nassar N. (2016). Planned birth before 39 weeks and child development: a population-based study. Pediatrics 138:e20162002. doi: 10.1542/peds.2016-2002, PMID: 27940704

[ref5] Bruce M. Ermanni B. Bell M. A. (2023). The longitudinal contributions of child language, negative emotionality, and maternal positive affect on toddler executive functioning development. Infant Behav. Dev. 72:101847. doi: 10.1016/j.infbeh.2023.101847, PMID: 37300924 PMC10527090

[ref6] Bruce M. McFayden T. C. Ollendick T. H. Bell M. A. (2022). Expressive language in infancyand toddlerhood: the roles of child temperament and maternal parenting behaviors. Dev. Psychobiol. 64:e22287. doi: 10.1002/dev.22287, PMID: 35748624 PMC9328282

[ref7] Buss A. H. Plomin R. (1984). Temperament: Early developing personality traits. Hillsdale, NJ: Erlbaum.

[ref8] Can D. D. Richards T. Kuhl P. K. (2013). Early gray-matter and white-matter concentration in infancy predict later language skills: a whole brain voxel-based morphometry study. Brain Lang. 124, 34–44. doi: 10.1016/j.bandl.2012.10.007 23274797 PMC3551987

[ref9] Dale P. S. Reznick J. S. Thal D. J. Marchman V. A. (2001). A parent report measure of language development for three-year-olds. Columbia, MO: University of Missouri-Columbia.

[ref10] Davis E. P. Buss C. Muftuler L. T. Head K. Hasso A. Wing D. A.. (2011). Children’s brain development benefits from longer gestation. Front. Psychol. 2:1. doi: 10.3389/fpsyg.2011.00001, PMID: 21713130 PMC3111445

[ref11] Davison L. Warwick H. Campbell K. Gartstein M. A. (2019). Infant temperament affects toddler language development. J. Educ. e-Learn. Res. 6, 122–128. doi: 10.20448/journal.509.2019.63.122.128

[ref12] Dhamrait G. K. Christian H. O’Donnell M. Pereira G. (2021). Gestational age and child development at school entry. Sci. Rep. 11:14522. doi: 10.1038/s41598-021-93701-y, PMID: 34267259 PMC8282628

[ref13] Dixon W. E. Jr. Shore C. (1997). Temperamental predictors of linguistic style during multiword acquisition. Infant Behav. Dev. 20, 99–103. doi: 10.1016/S0163-6383(97)90065-5

[ref14] Dixon W. E. Smith P. H. (2000). Links between early temperament and language acquisition. Merrill-Palmer Q. 46, 417–440.

[ref15] Eriksson M. Marschik P. B. Tulviste T. Almgren M. Pérez Pereira M. Wehberg S.. (2012). Differences between girls and boys in emerging language skills: evidence from 10 language communities. Br. J. Dev. Psychol. 30, 326–343. doi: 10.1111/j.2044-835X.2011.02042.x 22550951

[ref16] Espel E. V. Glynn L. M. Sandman C. A. Davis E. P. (2014). Longer gestation among children born full term influences cognitive and motor development. PLoS One 9:e113758. doi: 10.1371/journal.pone.0113758, PMID: 25423150 PMC4244187

[ref17] Fenson L. Marchman V. A. Thal D. Dale P. Reznick J. S. Bates E. (2007). MacArthur-Bates communicative development inventories: User's guide and technical manual. 2nd Edn. Baltimore, MD: Brookes Publishing Co.

[ref18] Foster-Cohen S. Edgin J. Champion P. Woodward L. (2007). Early delayed language development in very preterm infants: evidence from the MacArthur-Bates CDI. J. Child Lang. 34, 655–675. doi: 10.1017/S0305000907008070, PMID: 17822143

[ref19] Foster-Cohen S. H. Friesen M. D. Champion P. R. Woodward L. J. (2010). High prevalence/low severity language delay in preschool children born very preterm. J. Dev. Behav. Pediatr. 31, 658–667. doi: 10.1097/DBP.0b013e3181e5ab7e, PMID: 20613625

[ref9001] Frank M. C. Braginsky M. Yurovsky D. Marchman V. A. (2021). Variability and Consistency in Early Language Learning: The Wordbank Project. Cambridge, MA: MIT Press.

[ref20] Garello V. Viterbori P. Usai M. C. (2012). Temperamental profiles and language development: A replication and an extension. Infant Behav. Dev. 35, 71–82. doi: 10.1016/j.infbeh.2011.09.003, PMID: 22014745

[ref21] Gartstein M. A. Rothbart M. K. (2003). Studying infant temperament via the revised infant behavior questionnaire. Infant Behav. Dev. 26, 64–86. doi: 10.1016/S0163-6383(02)00169-8

[ref22] Germain N. Gonzalez-Barrero A. M. Byers-Heinlein K. (2022). Gesture development in infancy: effects of gender but not bilingualism. Infancy 27, 663–681. doi: 10.1111/infa.12469 35416417

[ref23] Goldsmith H. H. (1996). Studying temperament via construction of the toddler behavior assessment questionnaire. Child Dev. 67, 218–235. doi: 10.2307/1131697, PMID: 8605830

[ref24] Goldsmith H. H. Buss A. H. Plomin R. Rothbart M. K. Thomas A. Chess S.. (1987). Roundtable: what is temperament? Four approaches. Child Dev. 58, 505–529. doi: 10.2307/1130527, PMID: 3829791

[ref25] Hampson S. E. Goldberg L. R. (2006). A first large cohort study of personality trait stability over the 40 years between elementary school and midlife. J. Pers. Soc. Psychol. 91, 763–779. doi: 10.1037/0022-3514.91.4.763, PMID: 17014298 PMC2247365

[ref26] Hüppi P. S. Warfield S. Kikinis R. Barnes P. D. Zientara G. P. Jolesz F. A.. (1998). Quantitative magnetic resonance imaging of brain development in premature and mature newborns. Ann. Neurol. 43, 224–235. doi: 10.1002/ana.410430213 9485064

[ref27] Husby A. Wohlfahrt J. Melbye M. (2023). Gestational age at birth and cognitive outcomes in adolescence: population based full sibling cohort study. BMJ 380:e072779. doi: 10.1136/bmj-2022-072779, PMID: 36653028 PMC9846680

[ref28] Inder T. E. Warfield S. K. Wang H. Hüppi P. S. Volpe J. J. (2005). Abnormal cerebral structure is present at term in premature infants. Pediatrics 115, 286–294. doi: 10.1542/peds.2004-0326, PMID: 15687434

[ref29] Ishikawa-Omori Y. Nishimura T. Nakagawa A. Okumura A. Harada T. Nakayasu C.. (2022). Early temperament as a predictor of language skills at 40 months. BMC Pediatr. 22, 1–10. doi: 10.1186/s12887-022-03116-5 35062894 PMC8780364

[ref30] Kas B. Jakab Z. Lőrik J. (2022). Development and norming of the Hungarian CDI-III: A screening tool for language delay. Int. J. Lang. Commun. Disord. 57, 252–273. doi: 10.1111/1460-6984.12686, PMID: 34997807 PMC9304143

[ref31] Kas B. Lőrik J. Andrea S. V. Henrietta K. K. (2010). A korai nyelvi fejlődés új vizsgálóeszköze, a MacArthur-Bates Kommunikatív Fejlődési Adattár (KOFA) bemutatása és validitási vizsgálata. Gyógypedagógiai Szemle 38, 114–125.

[ref32] Keller K. Troesch L. M. Loher S. Grob A. (2016). The relation between effortful control and language competence—A small but mighty difference between first and second language learners. Front. Psychol. 7:1015. doi: 10.3389/fpsyg.2016.01015 27458410 PMC4933703

[ref33] Kopala-Sibley D. C. Olino T. Durbin E. Dyson M. W. Klein D. N. (2018). The stability of temperament from early childhood to early adolescence: A multi-method, multi-informant examination. Eur. J. Personal. 32, 128–145. doi: 10.1002/per.2151, PMID: 30858648 PMC6407883

[ref34] Kort B. Reilly R. Picard R. W. (2001). “An affective model of interplay between emotions and learning: reengineering educational pedagogy-building a learning companion” in Proceedings IEEE international conference on advanced learning technologies, 43–46.

[ref35] Laake L. M. Bridgett D. J. (2014). Happy babies, chatty toddlers: infant positive affect facilitates early expressive, but not receptive language. Infant Behav. Dev. 37, 29–32. doi: 10.1016/j.infbeh.2013.12.006, PMID: 24441013 PMC4267686

[ref36] Lakatos K. Tóth I. Gervai J. (2010). Csecsemő Viselkedési Kérdőív és Kora Gyermekkori Viselkedési Kérdőív Available at: https://research.bowdoin.edu/rothbart-temperament-questionnaires/instrument-descriptions/the-early-childhood-behavior-questionnaire/

[ref37] Law J. Clegg J. Rush R. Roulstone S. Peters T. J. (2019). Association of proximal elements of social disadvantage with children's language development at 2 years: an analysis of data from the children in focus (CiF) sample from the ALSPAC birth cohort. Int. J. Lang. Commun. Disord. 54, 362–376. doi: 10.1111/1460-6984.12442, PMID: 30479068

[ref38] Limperopoulos C. Soul J. S. Gauvreau K. Huppi P. S. Warfield S. K. Bassan H.. (2005). Late gestation cerebellar growth is rapid and impeded by premature birth. Pediatrics 115, 688–695. doi: 10.1542/peds.2004-1169, PMID: 15741373

[ref39] Lubsen J. Vohr B. Myers E. Hampson M. Lacadie C. Schneider K. C.. (2011). Microstructural and functional connectivity in the developing preterm brain. Semin. Perinatol. 35, 34–43. doi: 10.1053/j.semperi.2010.10.006, PMID: 21255705 PMC3063450

[ref40] Ma Q. Wang H. Rolls E. T. Xiang S. Li J. Li Y.. (2022). Lower gestational age is associated with lower cortical volume and cognitive and educational performance in adolescence. BMC Med. 20:424. doi: 10.1186/s12916-022-02627-3, PMID: 36329481 PMC9635194

[ref41] MacKay D. F. Smith G. C. S. Dobbie R. Pell J. P. (2010). Gestational age at delivery and special educational need: retrospective cohort study of 407,503 schoolchildren. PLoS Med. 7:e1000289. doi: 10.1371/journal.pmed.1000289, PMID: 20543995 PMC2882432

[ref42] Marinopoulou M. Billstedt E. Lin P. I. Hallerbäck M. Bornehag C. G. (2021). Number of words at age 2.5 years is associated with intellectual functioning at age 7 years in the SELMA study. Acta Paediatr. 110, 2134–2141. doi: 10.1111/apa.15835, PMID: 33686710

[ref43] Matte T. D. Bresnahan M. Begg M. D. Susser E. (2001). Influence of variation in birth weight within normal range and within sibships on IQ at age 7 years: cohort study. BMJ 323, 310–314. doi: 10.1136/bmj.323.7308.310, PMID: 11498487 PMC37317

[ref46] Nivins S. Kennedy E. McKinlay C. Thompson B. Harding J. E. Children with Hypoglycemia and Their Later Development (CHYLD) Study Team (2023). Size at birth predicts later brain volumes. Sci. Rep. 13:12446. doi: 10.1038/s41598-023-39663-9, PMID: 37528153 PMC10393952

[ref47] Özçalişkan Ş. Goldin-Meadow S. (2010). Sex differences in language first appear in gesture. Dev. Sci. 13, 752–760. doi: 10.1111/j.1467-7687.2009.00933.x, PMID: 20712741 PMC2923747

[ref48] Pérez-Pereira M. Fernández P. Resches M. Gómez-Taibo M. L. (2016). Does temperament influence language development? Evidence from preterm and full-term children. Infant Behav. Dev. 42, 11–21. doi: 10.1016/j.infbeh.2015.10.003, PMID: 26615329

[ref49] Posner M. I. Rothbart M. K. Voelker P. (2016). Developing brain networks of attention. Curr. Opin. Pediatr. 28, 720–724. doi: 10.1097/MOP.0000000000000413, PMID: 27552068 PMC5257020

[ref50] Putnam S. P. Gartstein M. A. Rothbart M. K. (2006). Measurement of fine-grained aspects of toddler temperament: the early childhood behavior questionnaire. Infant Behav. Dev. 29, 386–401. doi: 10.1016/j.infbeh.2006.01.004, PMID: 17138293 PMC4334385

[ref51] Putnam S. P. Helbig A. L. Gartstein M. A. Rothbart M. K. Leerkes E. (2014). Development and assessment of short and very short forms of the infant behavior questionnaire–revised. J. Pers. Assess. 96, 445–458. doi: 10.1080/00223891.2013.841171, PMID: 24206185

[ref52] Putnam S. P. Rothbart M. K. Gartstein M. A. (2008). Homotypic and heterotypic continuity of fine-grained temperament during infancy, toddlerhood, and early childhood. Infant Child Dev. 17, 387–405. doi: 10.1002/icd.582

[ref53] Roben C. K. P. Cole P. M. Armstrong L. M. (2013). Longitudinal relations among language skills, anger expression, and regulatory strategies in early childhood. Child Dev. 84, 891–905. doi: 10.1111/cdev.12027, PMID: 23278601 PMC3620964

[ref9002] Rogers C. E. Lean R. E. Wheelock M. D. Smyser C. D. (2018). Aberrant structural and functional connectivity and neurodevelopmental impairment in preterm children. J. Neurodev. Disord. 10, 1–13. 30541449 10.1186/s11689-018-9253-x PMC6291944

[ref54] Rothbart M. K. (2007). Temperament, development, and personality. Curr. Dir. Psychol. Sci. 16, 207–212. doi: 10.1111/j.1467-8721.2007.00505.x

[ref55] Rothbart M. K. Bates J. E. (2007). Temperament. In Handbook of child psychology. (eds.) Damon W. Eisenberg N. Vol. 3. Social, emotional, and personality development (New York: Wiley), pp. 105–176.

[ref56] Rothbart M. K. Derryberry D. (1981). “Development of individual differences in temperament” in Advances in Developmental Psychology. eds. Lamb M. E. Brown A. L., vol. 1 (Hillsdale, NJ: Lawrence Erlbaum), 37–86.

[ref57] Salley B. J. Dixon W. E. Jr. (2007). Temperamental and joint attentional predictors of language development. Merrill-Palmer Q. 53:7. doi: 10.1353/mpq.2007.0004 PMC2137170 18080005

[ref58] Shaw J. C. Berry M. J. Dyson R. M. Crombie G. K. Hirst J. J. Palliser H. K. (2019). Reduced Neurosteroid exposure following preterm birth and its’ contribution to neurological impairment: A novel avenue for preventative therapies. Front. Physiol. 10:599. doi: 10.3389/fphys.2019.00599, PMID: 31156466 PMC6529563

[ref59] Shiner R. Buss K. McClowry S. Putnam S. Saudino K. (2012). What is temperament now? Assessing Progress temperament research on the twenty-fifth anniversary of Goldsmith et al. Child Dev. Perspect. 6, 436–444. doi: 10.1111/j.1750-8606.2012.00254.x

[ref60] Shokrkon A. Nicoladis E. (2022). The directionality of the relationship between executive functions and language skills: a literature review. Front. Psychol. 13:848696. doi: 10.3389/fpsyg.2022.848696, PMID: 35928417 PMC9343615

[ref61] Snijders V. E. Bogicevic L. Verhoeven M. van Baar A. L. (2020). Toddlers’ language development: the gradual effect of gestational age, attention capacities, and maternal sensitivity. Int. J. Environ. Res. Public Health 17:7926. doi: 10.3390/ijerph17217926, PMID: 33137895 PMC7663656

[ref63] Stene-Larsen K. Brandlistuen R. E. Lang A. M. Landolt M. A. Latal B. Vollrath M. E. (2014). Communication impairments in early term and late preterm children: A prospective cohort study following children to age 36 months. J. Pediatr. 165, 1123–1128. doi: 10.1016/j.jpeds.2014.08.027, PMID: 25258153

[ref64] Stolt S. Haataja L. Lapinleimu H. Lehtonen L. (2009). The early lexical development and its predictive value to language skills at 2 years in very-low-birth-weight children. J. Commun. Disord. 42, 107–123. doi: 10.1016/j.jcomdis.2008.10.002, PMID: 19054524

[ref66] Tang A. Crawford H. Morales S. Degnan K. A. Pine D. S. Fox N. A. (2020). Infant behavioral inhibition predicts personality and social outcomes three decades later. Proc. Natl. Acad. Sci. USA 117, 9800–9807. doi: 10.1073/pnas.1917376117, PMID: 32312813 PMC7211953

[ref67] Thomas A. Chess S. (1977). Temperament and development. New York: New York University Press.

[ref71] Yang H. Yang S. Isen A. M. (2013). Positive affect improves working memory: implications for controlled cognitive processing. Cognit. Emot. 27, 474–482. doi: 10.1080/02699931.2012.713325, PMID: 22917664

[ref72] Ye Z. Zhou X. (2009). Executive control in language processing. Neurosci. Biobehav. Rev. 33, 1168–1177. doi: 10.1016/j.neubiorev.2009.03.003 19747595

