# Corrigendum: The influence of temperament and perinatal factors on language development: a longitudinal study

**DOI:** 10.3389/fpsyg.2024.1540286

**Published:** 2025-01-10

**Authors:** Andrea Balázs, Krisztina Lakatos, Veronika Harmati-Pap, Ildikó Tóth, Bence Kas

**Affiliations:** ^1^Institute for General and Hungarian Linguistics, HUN-REN Hungarian Research Centre for Linguistics, Budapest, Hungary; ^2^Sound and Speech Perception Research Group, Institute of Cognitive Neuroscience and Psychology, HUN-REN Research Centre for Natural Sciences, Budapest, Hungary; ^3^Department of Cognitive Science, Faculty of Natural Sciences, Budapest University of Technology and Economics, Budapest, Hungary; ^4^MTA-ELTE Language-Learning Disorders Research Group, Eötvös Loránd University, Bárczi Gusztáv Faculty of Special Needs Education, Budapest, Hungary

**Keywords:** language development, temperament, gestational age, longitudinal study, sex differences

In the published article, there was an error regarding the affiliation for Andrea Balázs. As well as having affiliations 1 and 2, they should also have 3. Department of Cognitive Science, Faculty of Natural Sciences, Budapest University of Technology and Economics, Budapest, Hungary.

In the published article, there was an error in the legend for Table 5 as published. Pearson's r or Spearman's rho, ^+^*p* < 0.10, ^*^p < 0.05, ^**^p < 0.01, ^***^p < 0.001. Bold: Correlation surviving Bonferroni correction (*p* < 0.05/5). The corrected legend appears below.

Pearson's r or Spearman's rho, ^+^*p* < 0.10, ^*^p < 0.05, ^**^p < 0.01, ^***^p < 0.001. Bold: highlights significant and trend-level correlations.

The authors apologize for these errors and state that this does not change the scientific conclusions of the article in any way. The original article has been updated.

